# Poly-γ-glutamic acid/chitosan nanogel greatly enhances the efficacy and heterosubtypic cross-reactivity of H1N1 pandemic influenza vaccine

**DOI:** 10.1038/srep44839

**Published:** 2017-03-21

**Authors:** Jihyun Yang, Sang-Mu Shim, Thi Quyen Nguyen, Eun-Ha Kim, Kwang Kim, Yong Taik Lim, Moon-Hee Sung, Richard Webby, Haryoung Poo

**Affiliations:** 1 Infectious Disease Research Center, Korea Research Institute of Bioscience and Biotechnology, Daejeon, Republic of Korea; 2 Viral Infectious Disease Research Center, Korea Research Institute of Bioscience and Biotechnology, Daejeon, Republic of Korea; 3 Department of Molecular Biology and the Institute for Molecular Biology and Genetics, Chonbuk National University, Jeonju, Republic of Korea; 4 Department of Biotechnology, University of Science and Technology, Daejeon, Republic of Korea; 5 College of Medicine, Chungbuk National University, Cheongju, Republic of Korea; 6 BioLeaders Corporation, Daejeon, Republic of Korea; 7 Sungkyunkwan Advanced Institute of Nanotechnology (SAINT) and Department of Chemical Engineering, Sungkyunkwan University Suwon, Republic of Korea; 8 Department of Bio and Fermentation Convergence Technology, Kookmin University, Seoul, Republic of Korea; 9 Division of Virology, Department of Infectious Diseases, St. Jude Children’s Research Hospital, Memphis, Tennessee, USA

## Abstract

In 2009, the global outbreak of an influenza pandemic emphasized the need for an effective vaccine adjuvant. In this study, we examined the efficacy of poly-γ-glutamic acid/chitosan (PC) nanogel as an adjuvant for the influenza vaccine. PC nanogel significantly enhanced antigen-specific cross-presentation and cytotoxic T lymphocyte (CTL) activity. Compared with alum, the protective efficacy of the pandemic H1N1 influenza (pH1N1) vaccine was substantially increased by PC nanogel, with increased hemagglutination-inhibition titers, CTL activity, and earlier virus clearance after homologous and heterosubtypic [A/Philippines/2/82 (H3N2)] virus challenges. However, CD8^+^ T cell-depleted mice displayed no protection against the heterosubtypic virus challenge after immunization with PC nanogel-adjuvanted pH1N1 vaccine. We also observed that using PC nanogel as a vaccine adjuvant had a dose-sparing effect and significantly enhanced the long-lasting protection of the pH1N1 vaccine. Together, these results suggest that PC nanogel is a promising vaccine adjuvant that could broadly prevent influenza virus infection.

Vaccination is considered to be the most effective strategy for controlling influenza infection. During pandemics, however, many countries have experienced vaccine shortages due to the relative lack of the manufacturing facilities needed and because of long and complicated vaccine production processes^1^,^2^. Such problems meant that the swine-origin H1N1 influenza A virus that emerged in 2009 and had high transmission efficiency among humans could not be effectively controlled, resulting in the global spread of the virus until 2010 and the infection of millions of people worldwide^3^. Clearly, we need to develop an effective vaccine strategy, such as the use of adjuvants that will decrease the required dose and enhance the efficacy of the influenza vaccine.

Aluminum compounds (e.g., aluminum hydroxide and aluminum phosphate) are already approved for use as vaccine adjuvants in humans^4^. Alum enhances antigen presentation by forming a complex at the injection site, increasing the local antigen concentration and improving uptake by antigen-presenting cells (APCs)^5^, and thereby significantly enhancing antibody production^6^. However, alum has little effect on cytotoxic T lymphocyte (CTL) responses. This feature is problematic because cell-mediated immune responses are important for protection against many pathogens, including viruses^7^. To date, most of the reported vaccine adjuvants induce robust antibody responses but trigger only limited cellular immune responses^8^,^9^,^10^. Therefore, we critically require an efficient adjuvant that is capable of inducing both humoral and cellular immune responses specific to the vaccine antigens.

Our group previously reported a robust strategy for synthesizing a nanogel adjuvant by combining two oppositely charged biocompatible polyelectrolytes originating from natural organisms, poly-γ-glutamic acid (γ-PGA) and chitosan, to create a novel γ-PGA/chitosan (PC) nanogel that triggered enhanced cellular and humoral immunity against a model antigen, ovalbumin (OVA)^11^. However, little is known about the effect of PC nanogel on cross-presentation of antigens by APCs or the triggering of CTL activity, even though both events are critical for successful vaccine-induced protection against viral infection^12^,^13^,^14^. Furthermore, the adjuvant effects of PC nanogel on homo- and heterosubtypic influenza virus challenges have not been investigated.

In this study, we compared the efficacies of PC nanogel and alum in inducing cross-presentation and CTL activity using the model antigen OVA. We then evaluated the adjuvant efficacies of PC nanogel and alum in enhancing the pandemic H1N1 influenza (pH1N1) hemagglutinin (HA)-specific cell-mediated immune responses, antibody production, and cross-protection against heterosubtypic influenza A virus in mouse and ferret models. Our results revealed that the PC nanogel-adjuvanted vaccine is more effective than the alum-adjuvanted vaccine in inducing cellular immune responses that confer cross-protection. Therefore, PC nanogel may be a promising vaccine adjuvant for the broad prevention of influenza virus infection.

## Results

### PC nanogel enhances cross-presentation and *in vitro* antigen-specific cellular immunity

**Figure 1:**
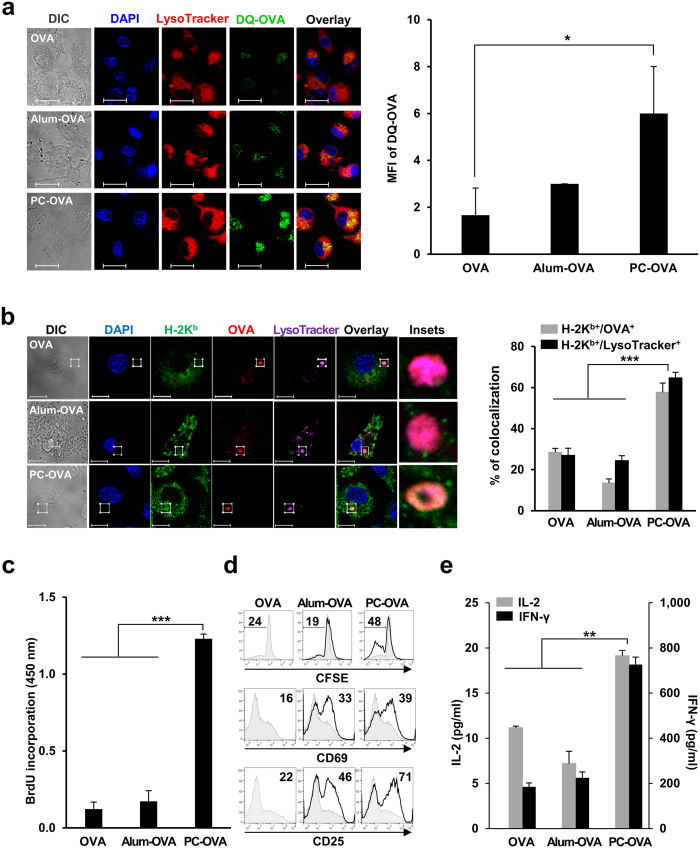
PC nanogel induces antigen uptake and phagosomal MHC-I enrichment of DCs, enhancing cross-presentation. (**a**) DCs were pulsed with DQ-OVA combined with an adjuvant (alum or PC nanogel) for 4 h followed by further incubating with LysoTracker, and observed by fluorescence microscopy. Scale bar, 20 μm. MFI per 20 cells was calculated using the LSM software. (**b**) DCs were incubated with OVA-Texas red plus alum or PC nanogel for 3 h followed by further incubating with LysoTracker. The cells were stained with fluorescent-conjugated anti-MHC-I and observed by fluorescence microscopy. Scale bar, 10 μm. Bar graphs indicate the percent of colocalization in the boxed insets (Manders’ coefficient; n = 20 cells). (**c**–**e**) DCs were pulsed with OVA combined with alum or PC nanogel for 6 h, washed, and then cocultured with OT-I CD8^+^ T cells. T cell proliferation was determined by (**c**) BrdU incorporation assays and (**d**) CFSE-labeled T cell division on day 3. The numbers in each histogram indicate the percentages of positive cells. The expressions of CD69 and CD25 were analyzed by flow cytometry at 12 h and 60 h post coculture, respectively. MFI is presented in each histogram. (**e**) The levels of IL-2 and IFN-γ in the culture supernatants were determined by ELISA. Statistical significance was analyzed by ANOVA/Bonferroni unless noted otherwise. *P <* *0.05; **P <* *0.01; and ***P <* *0.001. Data are presented representative of three independent experiments with similar results.

To investigate the effect of PC nanogel on antigen cross-presentation by APCs, we assessed antigen uptake and processing in murine bone marrow-derived dendritic cells (DCs) using DQ-OVA, which is OVA conjugated with boron-dipyrromethene (a self-quenched dye that emits green fluorescence upon degradation). The uptake of DQ-OVA and its colocalization with acidic compartments (lysosomes and phagolysosomes labeled with LysoTracker) inside DCs were measured by immunofluorescence confocal microscopy. Increased fluorescence inside acidic compartments of cells treated with PC nanogel-adjuvanted OVA (PC-OVA) was observed compared with those treated with OVA alone (OVA) or alum-adjuvanted OVA (alum-OVA). Quantitatively, DCs exposed to PC-OVA had significantly higher mean fluorescence intensities (MFIs) than DCs incubated with OVA or alum-OVA (*P* < 0.05) (Fig. 1a). A previous study has shown that cross-presentation is induced by the enrichment of MHC class I molecules (MHC-I) in phagosomes followed by enhanced formation of the antigen peptide-MHC-I complex^15^. To investigate the accumulation of MHC-I and the OVA antigen in the acidic compartments of DCs caused by PC nanogel, DCs were incubated with OVA-Texas red in the presence or absence of PC nanogel or alum. Then, we labeled MHC-I with anti-H-2K^b^ antibody and labeled acidic compartments with LysoTracker, followed by immunofluorescence confocal microscopy. The percentage colocalization of H-2K^b^ and OVA with acidic compartments was significantly greater in PC-OVA-treated DCs (H-2K^b^ and OVA, 57.71 ± 4.53%; H-2K^b^ and LysoTracker, 64.73 ± 2.74%) than in DCs exposed to OVA (H-2K^b^ and OVA, 28.43 ± 1.98%; H-2K^b^ and LysoTracker, 26.93 ± 3.53%) or alum-OVA (H-2K^b^ and OVA, 13.46 ± 2.05%; H-2K^b^ and LysoTracker, 24.38 ± 2.44%) (*P* < 0.001) (Fig. 1b). For cross-presentation, inhibitor of kappa B kinase 2 (IKK2) signaling is essential for the enhancement of the antigen peptide-MHC-I complex^15^. The PC nanogel-induced colocalization of H-2K^b^ and OVA with acidic compartments (H-2K^b^ and OVA, 51.2 ± 3.66%; H-2K^b^ and LysoTracker, 53.91 ± 4.21%) was significantly inhibited by IKK2 inhibitors such as BMS-345541 (H-2K^b^ and OVA, 14.62 ± 3.81%; H-2K^b^ and LysoTracker, 19.78 ± 4.32%), BAY11-7082 (H-2K^b^ and OVA, 14.75 ± 5.49%; H-2K^b^ and LysoTracker, 11.52 ± 3.93%), and TPCA-1 (H-2K^b^ and OVA, 12.32 ± 6.1%; H-2K^b^ and LysoTracker, 12.44 ± 6.2%). However, such effect was not observed by an ERK inhibitor, PD184352 (H-2K^b^ and OVA, 39.7 ± 9.45%; H-2K^b^ and LysoTracker, 43.87 ± 8.17%) (Supplementary Fig. 1). The PC nanogel also dose-dependently upregulated various costimulatory molecules (CD40, CD80, CD86, and MHC-II) (Supplementary Fig. 2a) and enhanced the production of proinflammatory cytokines (TNF-α, IL-1β, and IL-12) (Supplementary Fig. 2b), suggesting that PC nanogel increases the activation of DCs and cross-presentation by DCs, which increases CTL activity.

We assessed *in vitro* CTL activity using OT-I CD8^+^ T cells, which recognize an OVA_257–264_ peptide (SIINFEKL) presented by H-2K^b^ MHC-I^16^. OT-I CD8^+^ T cells were cocultured with DCs pulsed with PC-OVA or alum-OVA, and various proliferation and activation parameters of the CD8^+^ T cells were assessed. Optical density of bromodeoxyuridine (BrdU) incorporation was significantly increased in the OT-I CD8^+^ T cells cocultured with DCs that were exposed to PC-OVA (1.23 ± 0.03) versus those cocultured with DCs treated with OVA alone (0.14 ± 0.04) or with alum-OVA (0.17 ± 0.03) (*P* < 0.001) (Fig. 1c). The percentage of dividing cells in CFSE-labeled OT-I CD8^+^ T cells was also enhanced by coculture with DCs treated with PC-OVA (48%) compared with coculturing with DCs treated with OVA (24%) or alum-OVA (19%) (Fig. 1d). The surface expression levels of activation markers (CD69 and CD25) on OT-I CD8^+^ T cells were more strongly enhanced by coculture with DCs that had been exposed to PC-OVA versus those exposed to alum-OVA. Moreover, the production levels of IL-2 and IFN-γ were higher in OT-I CD8^+^ T cells cocultured with DCs that had been exposed to PC-OVA (IL-2, 19.12 ± 0.60 pg/mL; IFN-γ, 726.49 ± 32.65 pg/mL) compared with those of cells cocultured with DCs treated with OVA (IL-2, 11.16 ± 0.18 pg/mL; IFN-γ, 185.16 ± 16.96 pg/mL) or alum-OVA (IL-2, 7.18 ± 1.37 pg/mL; IFN-γ, 225.11 ± 25.74 pg/mL) (*P* < 0.01) (Fig. 1e). The increase in BrdU incorporation (Supplementary Fig. 3a) and IFN-γ production (Supplementary Fig. 3b) was significantly inhibited by IKK2 inhibitors but not by an ERK inhibitor in a dose-dependent manner. These results collectively indicate that PC nanogel enhances cross-presentation by DCs and induces the proliferation and activation of CD8^+^ T cells, thereby increasing antigen-specific CTL activity.

### PC nanogel augments *in vivo* antigen-specific cellular immunity

**Figure 2:**
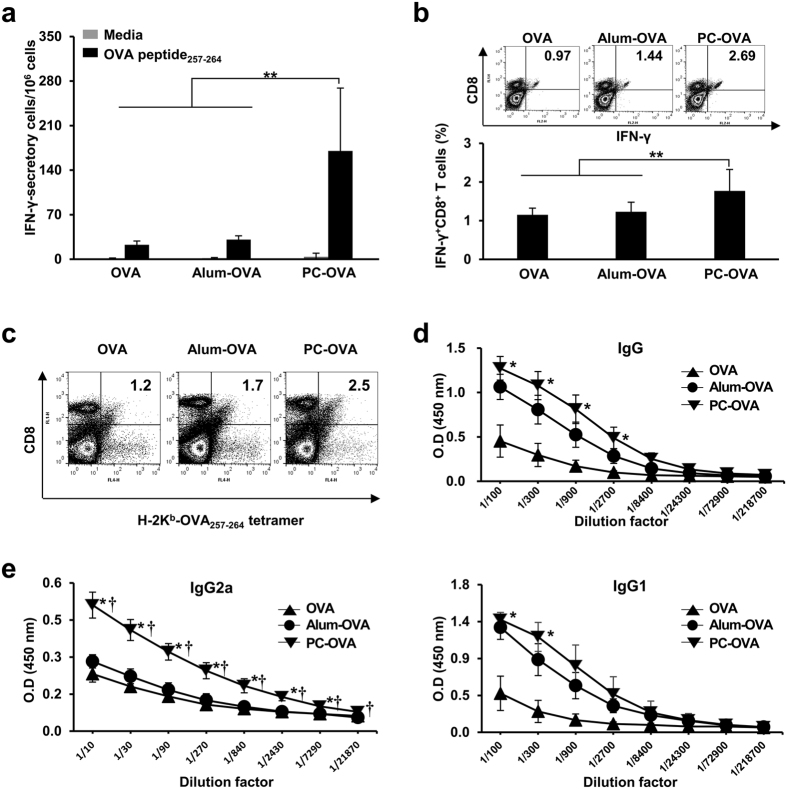
PC nanogel enhances antigen-specific cellular and humoral immunity. C57BL/6 mice (*n* = 5) were i.m. immunized on days 0, 14, and 21 with 10 μg of OVA mixed with alum or PC nanogel. Splenocytes were isolated, re-stimulated with OVA_257–264_ peptide, and subjected to (**a**) enumeration of IFN-γ-secreting cells by ELISPOT assay and (**b**) flow cytometric analysis for the percentages of IFN-γ-producing CD8^+^ T cells. (**c**) The generation of OVA_257–264_ peptide-specific (H-2K^b^-SIINFEKL) CD8^+^ T cells was determined in the splenocytes using flow cytometry. Statistically significant difference was analyzed by ANOVA/Bonferroni. *P < 0.05; **P < 0.01; and ***P < 0.001. (**d**,**e**) ELISA was used to test sera for OVA-specific antibodies, including (**d**) IgG, (**e**) IgG2a, and IgG1. Asterisk (*) and cross (†) indicate statistical significance at *P* < 0.001 when compared to the OVA alone group and the alum-OVA group, respectively. Data are presented representative of three independent experiments with similar results.

To evaluate the impact of PC nanogel on cellular and humoral immunity, mice were intramuscularly immunized with PC-OVA, alum-OVA, or OVA on days 0, 14, and 21. One week after the last immunization, we performed enzyme-linked immunosorbent spot (ELISPOT) assays and enumerated the OVA_257–264_-specific IFN-γ-secreting splenocytes. The number of the OVA_257–264_-specific IFN-γ-secreting splenocytes was significantly greater in mice immunized with PC-OVA (210 ± 96 spot forming units [SFUs]) compared with mice immunized with OVA (23 ± 3 SFUs) or alum-OVA (31 ± 3 SFUs) (Fig. 2a). Also, at 24 weeks after the final immunization, the increase of OVA_257–264_-specific IFN-γ-secreting splenocytes was persisted in OVA-PC group, whereas neither OVA nor alum-OVA group was shown such effect (Supplementary Fig. 4). In addition, the percentage of OVA-specific IFN-γ-secreting CD8^+^ T cells was significantly enhanced in the mice immunized with PC-OVA (1.91 ± 0.48%) compared with those immunized with OVA (1.17 ± 0.19%) or alum-OVA (1.28 ± 0.24%) (*P* < 0.01) (Fig. 2b). We labeled splenocytes with a fluorescent-conjugated H-2K^b^-OVA_257–264_ tetramer and an anti-CD8 antibody and assessed the cells by flow cytometry. The frequency of OVA_257–264_ tetramer-positive CD8^+^ T cells was significantly increased in mice treated with PC-OVA but not in mice immunized with OVA alone or alum-OVA (*P* < 0.01) (Fig. 2c). The level of OVA-specific IgG in the sera of mice immunized with PC-OVA was similar to that in the alum-OVA group (Fig. 2d). However, the level of the IgG2a isotype in the sera of mice in the PC-OVA group was significantly higher than that of mice immunized with alum-OVA (*P* < 0.001), whereas the IgG1 isotype levels were similar in these two groups (Fig. 2e). These results collectively indicate that PC nanogel augments antigen-specific cellular and humoral immune responses, predisposing the animals to Th1 responses.

### PC nanogel enhances the protective efficacy of the pH1N1 vaccine in mice

**Figure 3:**
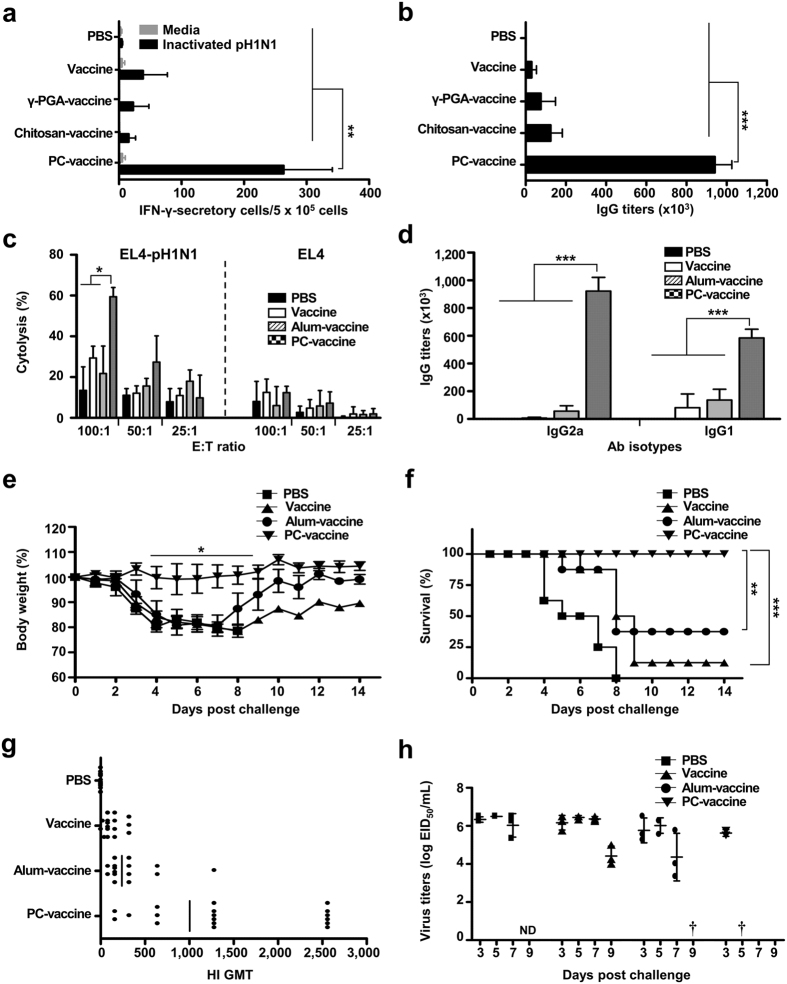
The use of PC nanogel as an adjuvant enhances the protective efficacy of the influenza vaccine in mice. C57BL/6 mice (*n* = 8) were i.m. immunized two times with 0.2 μg of pandemic vaccine at 0 and 14 days. At 28 days after the initial immunization, (**a**) vaccine antigen-specific-IFN-γ-producing splenocytes and (**b**,**d**) antibody titers were detected by ELISPOT assays and ELISA, respectively. (**c**) Cytolytic activity among EL4 target cells subjected to viral or mock infections at the indicated E:T ratios, as assessed by lactate dehydrogenase assay. (**e**–**h**) The vaccinated mice were challenged with 10 LD_50_ A/California/04/09 (CA04) virus. (**e**) Body weight changes and (**f**) survival rates were monitored for up to 14 days post-challenge. Each data point represents an average percentage. (**g**) Serum HI antibody titers against a homologous virus were measured. Results are shown as individual and HI GMT titers. (**h**) Viral titers were measured from mouse lungs after challenge (ND, not detected; †, virus clearance), and are presented as the mean value (±SD) of log_10_EID_50_/mL. Statistical significance was analyzed by ANOVA/Bonferroni, except (**f**) by log-rank test. *P <* *0.05; **P <* *0.01; and ***P <* *0.001.

To validate the efficacy of PC nanogel as a vaccine adjuvant, we immunized mice twice (day 0 and day 14) with the pH1N1 split vaccine antigen (A/California/7/2009 NYMC X-179A H1N1) mixed with PC nanogel (PC-vaccine), γ-PGA (γ-PGA-vaccine), or chitosan (chitosan-vaccine). Mice immunized with vaccine antigen alone or that underwent mock immunization were used as negative controls. Our results revealed that the number of IFN-γ-secreting splenocytes was robustly increased in the PC-vaccine group (263 ± 78 SFUs), but not in the γ-PGA- (22 ± 25 SFUs) or chitosan-vaccine groups (15 ± 11 SFUs), compared with the negative controls (*P* < 0.01) (Fig. 3a). The pH1N1-specific IgG antibody titer was significantly higher in mice injected with the PC-vaccine than in those immunized with the γ-PGA- or chitosan-vaccines or the antigen-only control (Fig. 3b). We next immunized mice with the pH1N1 vaccine antigen treated with an adjuvant of either PC nanogel (PC-vaccine) or alum (alum-vaccine) and then compared the pH1N1-specific CTL activity to determine whether an effective cell-mediated response was generated. More pH1N1-specific CTL activity was induced when PC nanogel was used as the adjuvant (59.4%) than when alum was used (21.7%) (*P* < 0.05) (Fig. 3c). Immunization with the PC-vaccine induced greater increases in the titers of the various IgG subclasses compared with the alum-vaccine immunization. The IgG2a (922,829 ± 98,805) and IgG1 (584,647 ± 63,056) isotype titers were both strikingly higher in the PC-vaccine group than in either the alum-vaccine (IgG2a, 56,614 ± 38,592; IgG1, 136,268 ± 78,531) or vaccine control (IgG2a, 6,913 ± 5,957; IgG1, 81,753 ± 98,546) groups (Fig. 3d).

To examine the protective efficacy, mice were vaccinated with PC-vaccine, alum-vaccine, or vaccine antigen alone (vaccine), allowed to establish a vaccine response for 2 weeks, and then challenged with a lethal dose (10 LD_50_) of the pH1N1 virus (A/California/04/09). Mice immunized with vaccine or the alum-vaccine were partially protected, exhibiting survival rates of 12.5% and 37.5%, respectively, whereas all the mice in the mock-vaccinated group (PBS) died. In contrast, no mouse that received the PC-vaccine died or lost body weight (Fig. 3e,f). This finding demonstrates that the PC nanogel adjuvant effectively induces protective immunity against the homologous virus.

To confirm the efficacy of the PC-vaccine, we examined hemagglutination inhibition (HI) titers, which have been correlated with the protective efficacy of influenza vaccines^17^, in mice immunized with an alum-vaccine or PC-vaccine. The HI antibody titers against the pH1N1 virus elicited by the PC-vaccine were 4-fold higher than those elicited by the alum-vaccine (1,004.27 geometric mean titer [GMT] versus 239.01 GMT, respectively) (Fig. 3g). Because clearance of the remaining virus from the lung is an important indicator of a vaccine’s protective efficacy, we determined the viral titers in lung extracts sampled on the indicated days after a viral challenge. Our results revealed that the PC-vaccine group was significantly better at clearing the pH1N1 virus. On day 5 post-challenge, the PC-vaccine group exhibited clearance, whereas the vaccine and alum-vaccine groups still had high viral loads (6.44 ± 0.1 EID_50_/mL and 6.01 ± 0.4 EID_50_/mL, respectively). Viral titers were detected in the alum group on day 7, and the virus had not yet been cleared in the vaccine-only group by day 9 (Fig. 3h). Collectively, these results indicate that the PC-vaccine induces the most effective protection against the pH1N1 virus in mice.

Previously, we reported that TLR4 signaling is essential for γ-PGA-induced DC activation^18^. Herein, we found that our γ-PGA-based PC nanogel induced minimal maturation of or antigen uptake by TLR4-deficient DCs (Supplementary Fig. 5a,b). Moreover, the number of IFN-γ-secreting cells in TLR4-deficient mice treated with the PC-vaccine was attenuated compared with that obtained in wild-type mice and was similar to that observed in mice immunized with vaccine alone (*P* < 0.001) (Supplementary Fig. 5c). Additionally, the PC-vaccine triggered less antibody production in TLR4-deficient mice than in wild-type mice (Supplementary Fig. 5d,e). These findings indicate that our PC nanogel exhibits adjuvant efficacy for the pH1N1 vaccine, in particular for increasing CTL activity through TLR4 signaling.

### PC nanogel enhances the protective efficacy of the pH1N1 vaccine in ferrets

**Figure 4:**
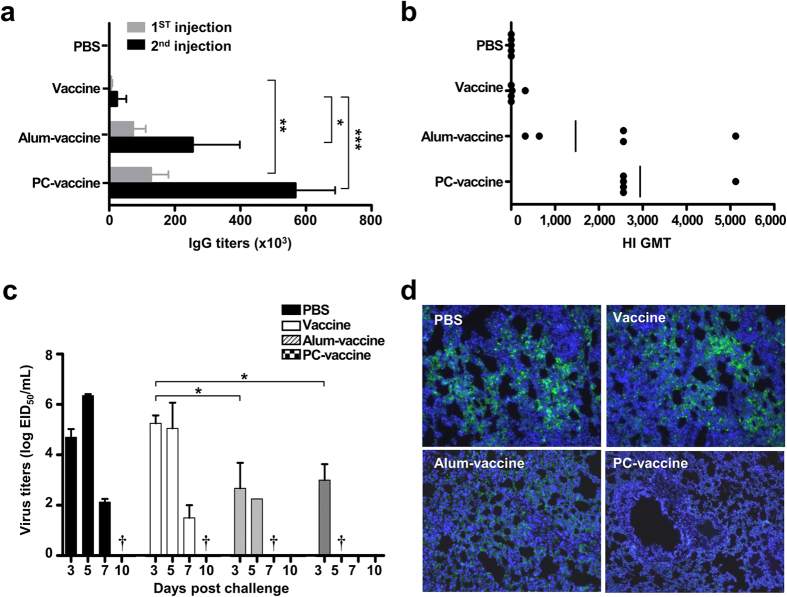
The use of PC nanogel as an adjuvant enhances the protective efficacy of the influenza vaccine in ferrets. Vaccinated ferrets (*n* = 5) were analyzed for antibody titers and challenged with A/California/04/09 (CA04) virus. (**a**) ELISA was used to test sera for vaccine antigen-specific antibody titers. (**b**) Serum HI antibody titers were measured against the homologous virus. Results are shown as individual and HI GMT titers. (**c**) Viral titers in nasal wash samples were measured from each group after challenge (†, virus clearance), and are presented as the mean (±SD) of log_10_EID_50_/mL. (**d**) To test for the presence of the infected viruses, lung tissues were stained with FITC-conjugated anti-influenza NP antibody (green), and nuclei were stained with DAPI (blue). Statistical significance was analyzed by ANOVA/Bonferroni for (**a**) or *t*-test for (**c**). *P < 0.05; **P < 0.01; and ***P < 0.001. Data are presented representative of two independent experiments with similar results.

Because ferrets are considered to be the most suitable animal model for the preclinical evaluation of human influenza vaccines^19^, we tested the efficacy of the PC nanogel adjuvant in a ferret model. Immunization with the PC-vaccine resulted in a higher IgG titer than did immunization with either the alum-vaccine or vaccine alone (Fig. 4a). Moreover, when HI antibody titers were measured using serum samples obtained 2 weeks after the final immunization, the GMT of HI antibodies in the PC-vaccine group was 2-fold higher than that observed in the alum-vaccine group (2,940.67 versus 1,470.33, respectively) (Fig. 4b). After the vaccinations, the ferrets were infected intranasally with pH1N1 virus, and the replicating virus titers in the upper respiratory tract were determined at various time points. In the PC-vaccine group, the mean value of the nasal wash titers was 213-fold lower than that in the vaccine-only group on day 3 post-challenge, with clearance observed on day 5. However, the alum-vaccine group exhibited viral clearance on day 7. The vaccine and PBS groups continued to shed virus on day 7 post-challenge (Fig. 4c). Furthermore, using lung sections obtained 5 days after the viral challenge, staining with FITC-conjugated anti-influenza A nucleoprotein (NP) antibodies detected the presence of influenza virus in the alum-vaccine and control groups but not in the PC-vaccine group (Fig. 4d). These data show that PC nanogel used as an adjuvant enhances virus-specific immune responses and inhibits viral replication in the upper and lower respiratory tracts more rapidly than alum.

### PC nanogel allows dose sparing of the pandemic vaccine and confers long-term protective immunity

**Figure 5:**
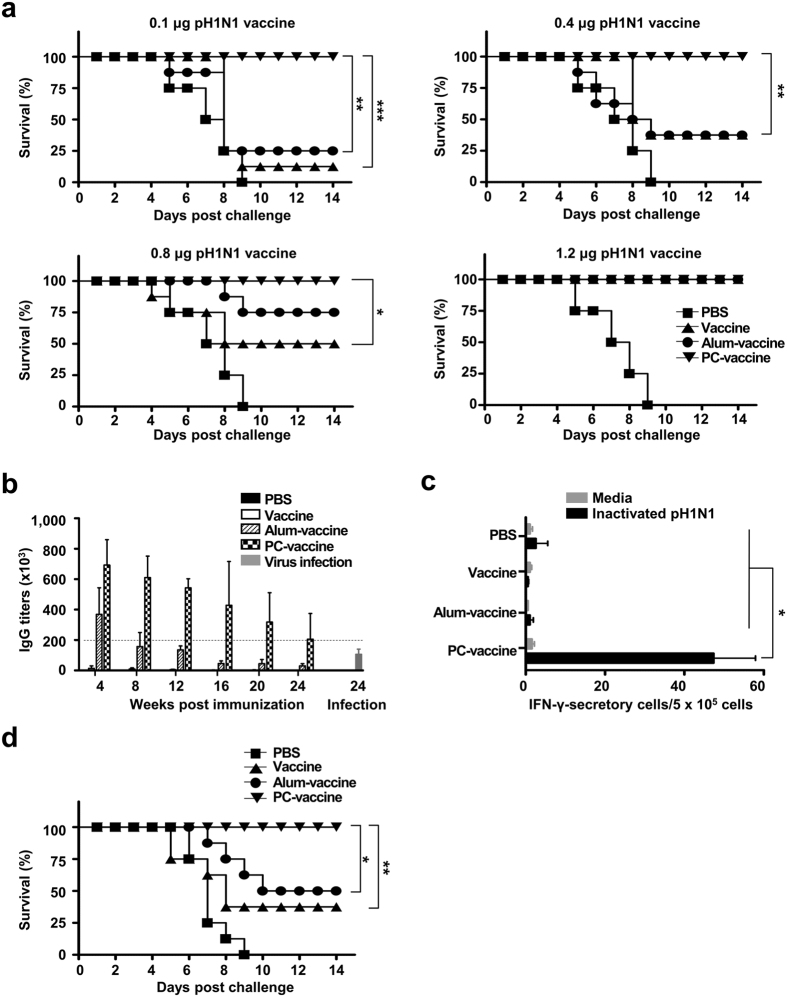
PC nanogel induces a vaccine dose-sparing effect and long-term protective immunity. (**a**) The survival rates of mice (*n* = 8) were monitored for up to 14 days after vaccination with pandemic vaccines containing 0.1, 0.4, 0.8, or 1.2 μg of HA along with the same dose of an adjuvant (alum or PC nanogel). Each data point represents an average percentage. (**b**–**d**) C57BL/6 mice (*n* = 8) were immunized twice with 0.2 μg of adjuvanted pandemic vaccine and then challenged with 10 LD_50_ of A/California/04/09 virus 24 weeks after the final immunization. (**b**) ELISA was used to test sera for pandemic vaccine antigen-specific IgG antibody titers, which are shown as the GMT (±SD). The sera of mice infected with a sub-lethal dose of pH1N1 virus were obtained at 24 weeks post challenge. (**c**) Splenocytes were collected 24 weeks after the final immunization, prior to viral challenge. The number of IFN-γ-producing T cells was detected by ELISPOT assays. (**d**) Survival rates were monitored for 14 days after the viral challenge. Each data point represents an average percentage. Statistical significance was analyzed by log-rank test for (**a**,**d**) or ANOVA/Bonferroni for (**c**). *P < 0.05; **P < 0.01; and ***P < 0.001. Data are presented representative of two independent experiments with similar results.

To evaluate the dose-sparing effect of using PC nanogel as an adjuvant for the influenza vaccine, we examined the efficacy of PC nanogel combined with various doses (0.1 to 1.2 μg/mice) of vaccine antigen. All the animals in the PC-vaccine groups survived without body weight loss, whereas the highest tested vaccine dose (1.2 μg/mice) was required for 100% survival in the vaccine and alum-vaccine groups (Fig. 5a). These results indicate that PC nanogel confers a dose-sparing effect, yielding effective protective immunity at a dose 12-fold lower than the dose required in either the alum-vaccine or vaccine alone groups.

To determine the ability of the PC-vaccine to confer long-term immunity against a lethal dose of the pandemic virus, we first tested sera from immunized mice for HA-specific humoral and cellular immune responses at 24 weeks after the final immunization. Only the PC-vaccine group had HA-specific antibody titers higher than those induced by infection with a sub-lethal dose of the virus (Fig. 5b). We then examined the pH1N1 virus-specific, cell-mediated immune response over time by analyzing the presence of IFN-γ-secreting cells 24 weeks after the final immunization. The numbers of IFN-γ-secreting T cells were higher in the PC-vaccine group (47.33 ± 10.61 SFUs) than in the alum-vaccine group (1.00 ± 0.94 SFUs), suggesting that pandemic H1N1 virus-specific T-cell memory was induced by the PC-vaccine (Fig. 5c). Finally, mice were challenged with the pandemic virus 24 weeks after the final immunization, and survival rates were monitored daily for 14 days post-challenge. Only the PC-vaccine group exhibited a 100% survival rate, whereas the alum-vaccine group had a survival rate of 37% (Fig. 5d). These results indicate that immunization with the PC-vaccine induces long-lasting protective immunity against the pH1N1 virus, including both humoral and cellular immune responses that react promptly to subsequent viral infections.

### PC nanogel enhances the cross-protective efficacy of the pH1N1 vaccine

**Figure 6:**
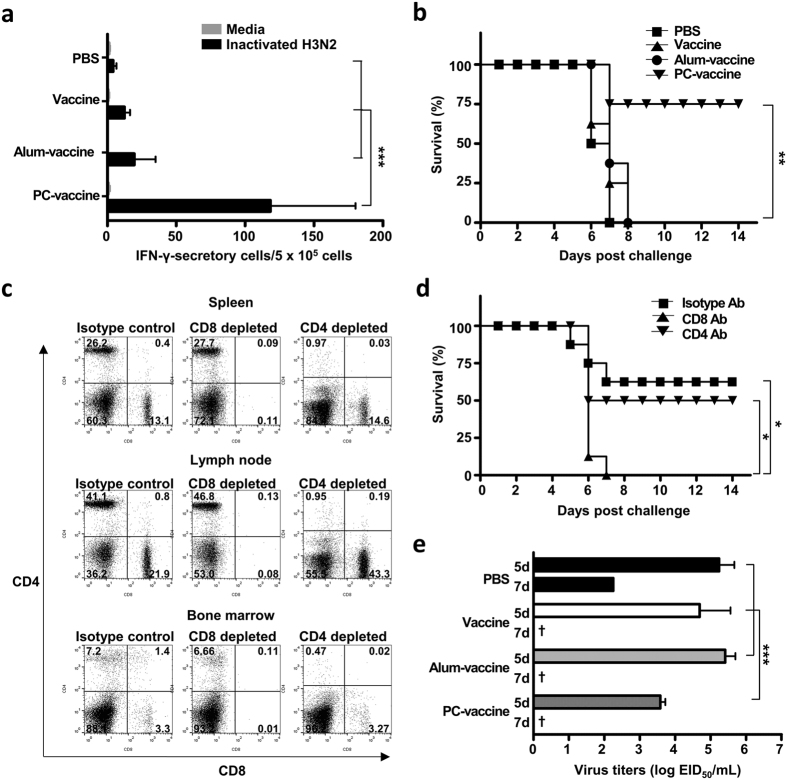
PC nanogel enhances the heterosubtypic cross-protective efficacy of the influenza vaccine in mice and ferrets. C57BL/6 mice (*n* = 8) and ferrets (*n* = 3) were given pandemic vaccinations two times on days 0 and 14. At 28 days after the initial vaccination, (**a**) the splenocytes were harvested from the mice and stimulated with inactivated H3N2 virus, and IFN-γ-producing T cells were assessed by ELISPOT assays. (**b**) The survival rates of mice were monitored for up to 14 days after challenge with 5 LD_50_ H3N2. (**c**,**d**) Before heterologous challenge, PC-vaccine-immunized mice received three times antibody-mediated depletion treatments. (**c**) Efficacy of CD8^+^ or CD4^+^ cells depletion was confirmed by flow cytometry. (**d**) At one day after the final administration of depletion antibodies, the mice were challenged with 5 LD_50_ H3N2 for monitoring survival rates. Each data point represents an average percentage. (**e**) Vaccinated ferrets were challenged either mock or 1 mL of 10^6^ EID_50_/mL of H3N2 virus, and nasal wash samples were collected on days 5 and 7 post-challenge. Viral titers are presented as the mean (±SD) of the log_10_ EID_50_/mL. Statistical significance was analyzed by ANOVA/Bonferroni for (**a**,**e**) or Log-rank test for (**b**,**d**). *P < 0.05; **P < 0.01; and ***P < 0.001. Data are presented representative of two independent experiments with similar results.

To determine the cross-protective efficacy of PC nanogel, mice were immunized two times with pH1N1 vaccine mixed with either PC nanogel or alum on days 0 and 14. At 28 days after the initial immunization, splenocytes of the vaccinated mice were harvested and stimulated with UV-inactivated H3N2 virus, and IFN-γ secretory cells were assessed by ELISPOT assay. Compared with the number of IFN-γ-secreting splenocytes in the PBS control group (4.11 ± 2.50 SFUs), the PC-vaccine group had significantly more of these cells (118.33 ± 61.75 SFUs), whereas there was no significant change in the vaccine (12.33 ± 4.10 SFUs) or alum-vaccine (19.44 ± 15.50 SFUs) groups (Fig. 6a). On day 28 after the initial immunization, the mice were challenged with a lethal dose (5 LD_50_) of H3N2 virus. The survival rate of the PC nanogel group was 75%, which was significantly higher than that of the other groups (0% survival) at day 8 after the viral challenge (Fig. 6b). However, the results of the HI assays with the H3N2 virus revealed that antibodies present in the pandemic vaccine-immunized sera had no cross-HI reactivity with the heterosubtypic H3N2 virus (Supplementary Fig. 6), suggesting that neutralizing humoral immunity did not play a role in the protection against heterosubtypic H3N2 virus infection in the PC-vaccine group.

To investigate whether the cross-protection induced by the PC-vaccine was mediated by CD8^+^ CTLs, we repeated the vaccination/infection experiments using CD8^+^ or CD4^+^ cell-depleted mice. Flow cytometry demonstrated that anti-CD8 or anti-CD4 antibodies treatment reduced the percentages of CD8^+^ cells (spleen, 13.1 to 0.11%; lymph node, 21.9 to 0.08%; bone marrow, 3.3 to 0.01%) or CD4^+^ cells (spleen, 26.2 to 0.97%; lymph node, 41.1 to 0.95%; bone marrow, 7.2 to 0.47%), respectively (Fig. 6c). Whereas the control group (isotype-matching antibody-injected) was partially protected (62.5% survival) against the H3N2 virus, the CD8^+^ T cell-depleted group was completely unprotected (0% survival) in the PC-vaccine group. In contrast, when CD4^+^ cell-depleted mice were used, the PC-vaccine group was partially protected (50% survival) against the heterosubtypic virus (Fig. 6d). Thus, the cross-protection provided by the PC-vaccine against heterosubtypic challenge appears to be mediated by CD8^+^ T cells.

In the ferret model, we examined the replicating virus titers in the upper respiratory tract of ferrets immunized with an alum-vaccine or PC-vaccine after an H3N2 virus challenge. Our results revealed that the PC-vaccine group had significantly lower replicative virus titers on day 5 compared with the vaccine-only or alum groups, which exhibited high viral loads (Fig. 6e). Collectively, these results suggest that PC nanogel dramatically enhances the heterosubtypic cross-protection activity of the pH1N1 vaccine against H3N2 virus infection in both mouse and ferret models.

## Discussion

Here we report the use of a biocompatible polyelectrolyte-based nanogel composed of γ-PGA and chitosan (PC nanogel) to deliver vaccine antigens and assess its use as an adjuvant for the influenza vaccine. Other γ-PGA-containing particles have been used for similar purposes^20^,^21^,^22^,^23^, because γ-PGA is a biodegradable and edible polymer produced by *B. subtilis*^24^,^25^. For example, intraperitoneal immunization with the Japanese encephalitis (JE) vaccine plus γ-PGA-graft-L-phenylalanine (L-Phe) nanoparticles (NPs) was shown to induce an immune response that protected mice against challenge with the JE virus^26^. Moreover, subcutaneous^20^ or intranasal^27^ immunization with γ-PGA-graft-L-Phe NPs plus the inactivated influenza HA antigen enhanced virus-specific humoral and cell-mediated immune responses more efficiently than a more complex system in which HA was attached to or packaged in the γ-PGA-graft-L-Phe NPs. These NPs were self-assembled by manipulating the hydrophobic-hydrophilic balance; to enable this assembly, the authors chemically conjugated the hydrophobic L-Phe molecule onto the carboxylic acid groups of γ-PGA^26^. Although γ-PGA-graft-L-Phe NPs have performed well as adjuvants, more systematic research is warranted, and researchers should seek to develop safer and more effective materials for human use^28^. Therefore, we avoided the use of synthetic grafted polymers in making the PC nanogel. Instead, we chose γ-PGA, which activates APCs including macrophages and DCs^18^,^29^, and chitosan, which offers the benefits of low immunogenicity and good biocompatibility and biodegradability and has been widely used as a potent carrier for drug delivery^30^,^31^. Thus, our PC nanogel is degradable by γ-glutamyl transpeptidase, which is distributed throughout the human body^32^,^33^. Using the model antigen OVA, γ-PGA nanomaterials have been shown to associate with antigens and increase the uptake of these antigens by APCs, especially DCs, thereby enhancing antigen-specific cellular and humoral immune responses^11^,^34^.

In this study, we first used OVA as a model antigen to compare the adjuvant effects of PC nanogel versus alum, a commercial vaccine adjuvant approved for human use. Our results revealed that, unlike alum, PC nanogel efficiently induced cross-presentation through increases in both antigen uptake/processing and MHC-I/antigen complex formation with OVA as the model antigen, thereby triggering CTL activity. CTLs typically express CD8 and induce the apoptosis of cells on which MHC-I molecules present recognizable foreign antigens^12^. Furthermore, the cross-presentation of exogenous antigens on the MHC-I molecules of DCs is essential for the initiation of CD8^+^ T-cell immune responses^35^,^36^,^37^. Compared with alum-OVA, PC-OVA was more efficiently engulfed and processed in DCs, where the PC-OVA induced more robust DC activation. Compared with alum, the use of PC nanogel as an adjuvant also enhanced the interaction of MHC-I and OVA within acidic compartments, such as phagolysosomes and lysosomes, leading to cross-presentation. These results are consistent with a previous report showing that the induction of cross-presentation is mediated by phagosomal MHC-I enrichment^15^.

Our next set of experiments showed for the first time that using PC nanogel as an adjuvant for the pandemic influenza vaccine antigen enhanced the virus-specific, cell-mediated immune response and the production of neutralizing antibodies. Compared with the alum-vaccine, the PC-vaccine was also significantly more effective at inducing protective immunity and viral clearance against heterosubtypic influenza virus infection in our mouse and ferret models. The depletion of CD8^+^ T cells completely inhibited the PC nanogel-induced heterosubtypic cross-reactivity of the pandemic vaccine, indicating that PC nanogel-enhanced cytolytic activities play a major role in the cross-protection obtained against the heterosubtypic challenge. Because influenza A viruses exist as several subtypes, the use of adjuvants capable of enhancing heterosubtypic cross-protection is critical to the efficacy of the influenza vaccine. Heterosubtypic immunity is primarily mediated by CTLs, which recognize conserved protein epitopes that are shared across influenza A virus subtypes^14^,^38^. Consistent with this concept, we found that the PC nanogel-mediated induction of heterosubtypic immunity depends on CTL activity. The PC nanogel-induced enhancement of CTL responses may accelerate the inhibition of heterosubtypic virus replication in the respiratory tract. These findings indicate that our PC nanogel induces cross-presentation, thereby increasing CTL activity. Further studies may be required to completely understand the mechanism of the cross-protective efficacy of PC nanogel.

Because limited influenza vaccine production has been a major problem during influenza pandemics, the World Health Organization has called for the development of vaccine-adjuvant pairs that can improve immunogenicity while sparing the antigen and inducing protective immunity^39^,^40^. In this study, we demonstrated that PC nanogel is an excellent adjuvant that enhances both cellular and humoral immunity and, when paired with the pandemic influenza vaccine antigen, induces 100% protection against challenge by the 2009 H1N1 virus. PC nanogel enabled us to use a smaller effective dose of the vaccine. This finding might be important for the development of a future pandemic vaccine when an influenza pandemic occurs during a vaccine shortage. Moreover, although the goal for vaccination is to induce long-term protective immunity, including a memory response^41^, the currently available inactivated influenza vaccine does not induce long-lasting immune responses^42^. We herein reported that the PC nanogel induced long-lasting protective immunity. Intramuscular vaccination with the PC-vaccine induced virus-specific antibodies, and the cell-mediated responses persisted throughout the 6-month analysis period. Moreover, mice immunized with the PC-vaccine were completely protected against a lethal challenge with the pandemic influenza virus at 6 months post-vaccination. Recent reports have shown that CD8^+^ T cell immunity is important for the efficacy of vaccination in the elderly^43^,^44^ because such individuals can induce functional memory CD8^+^ T cells^45^,^46^. Based on our finding that the PC nanogel significantly increased CD8^+^ T cell-mediated immunity and long-term protection, we propose that it could prove useful as an adjuvant for influenza vaccines, especially for the elderly.

Until now, various kinds of adjuvants have been developed after aluminum salts and emulsion were approved for human vaccines^47^,^48^,^49^,^50^. Aluminum salts consist of crystalline nanoparticles that aggregate to form a heterogeneous dispersion of micron-sized particles and have been employed as adjuvants in human vaccines for many decades^47^,^48^. Other formulations vaccine adjuvants such as liposomes and polymeric nanoparticles have been also developed and tested in preclinical fields^50^,^51^. Compared with previous vaccine adjuvants, the recent development of next-generation adjuvants have been rationally designed based on the insights into the innate immune system and its importance in initiating the adaptive immune response^52^,^53^. They have adopted one class of pattern recognition receptors (PRRs) called TLRs as vaccine adjuvant targets^53^. PC nanogel also induced innate immunity mediated by TLR4 that facilitate the generation of T cell responses. The most commonly used adjuvants in vaccines approved for human use, including alum and oil-in-water emulsion–based adjuvants, are not optimal for the targeting of T cell responses. In contrast, PC nanogel significantly enhanced antigen-specific cross-presentation and CTL activity. For the clinical application, most of nanoparticle-based adjuvants have potential limitations such as insufficient efficacy, difficult manufacturing, unacceptable toxicity, and economic feasibility. PC nanogel adjuvated vaccine induced earlier virus clearance after heterosubtypic virus challenges in mice model, which had not been reported in most of other nanoparticle-based adjuvants containing poly(lactide-co-glycolide), polyanhydrides, polypropylene sulfide and liposome^54^,^55^,^56^. In the fabrication point of view, the PC nanogels were easily manufactured by the electrostatic interaction between two bio-derived polymeric components, γ-PGA and chitosan^11^. Unlike nanoparticle-based adjuvants sensing by TLR ligands (i.e. CpG and imidazoquinolines) that have been limited due to systemic toxicity and side effects^57^, PC nanogel is composed of relatively safe components, γ-PGA and chitosan. The safety of PC nanogel would facilitate the translation them into clinics. Furthermore, PC nanogel is more cost-effective than chemically synthetic nanoparticles including PLGA and liposomes incorporated with TLR agonists because it was manufactured without chemical conjugation and the use of expensive TLR ligands.

In this report, we showed that influenza vaccine antigen-specific adaptive immune responses (e.g., antibody production and CTL activity) were significantly increased by the coadministration of the vaccine with the PC nanogel adjuvant in animal models. The PC-vaccine elicited high HI antibody responses and efficiently protected against viral challenge, and the use of PC nanogel conferred a dose-sparing effect. The PC nanogel potently induced cross-presentation by enhancing antigen uptake and promoting the MHC-I-antigen interaction. Furthermore, our results indicate that the heterosubtypic cross-reactive immunity triggered by the PC nanogel adjuvant may be mediated by CTL activity. Finally, the administration of a PC-vaccine induced a long-lasting protective immunity against the homosubtypic pH1N1 virus. Our results collectively suggest that PC nanogel may be a promising adjuvant for vaccines targeted against pandemic influenza and other infectious diseases.

## Methods

### Animals and cells

Six- to eight-week-old female C57BL/6 and OT-I transgenic mice were purchased from Koatech and Jackson Laboratory, respectively. Three- to eight-month-old ferrets were purchased from Marshall BioResources. All animal experiments were reviewed and approved by the Institutional Animal Care and Use Committee (IACUC) of the Korea Research Institute of Bioscience and Biotechnology (KRIBB) and were performed according to the Guidelines for Animal Experiments of the KRIBB. Animals were housed in a specific pathogen-free facility in the KRIBB. Bone marrow-derived DCs were generated as previously described^18^. Mouse EL4 cells were purchased from the American Type Culture Collection. All cells were maintained in RPMI 1640 containing 10% heat-inactivated FBS, 100 U/mL penicillin, and 100 mg/mL streptomycin (Gibco).

### Vaccine antigens and PC nanogel

The pH1N1 split vaccine antigen (A/California/7/2009 NYMC X-179A H1N1) was kindly provided by Mogam Biotechnology Research Institute. PC nanogel was produced by coprecipitating γ-PGA (BioLeaders Corporation) and chitosan (CNA BIOTECH) dissolved in a 0.85% saline solution. Briefly, chitosan solution (1.2 mg/mL) was added drop-wise to γ-PGA solution (1.2 mg/mL), with constant stirring at 70 × *g*. The resulting PC nanogel was collected by centrifugation at 300 × *g* for 30 min and resuspended in a 0.85% saline solution. The size of the nanogel particles was measured as indicated previously^11^ and the average size of those was 283 ± 50 nm. The degree of bacterial endotoxin contamination in the PC nanogel was determined using Limulus amebocyte lysate assay and was less than 10 endotoxin units per mL.

### Viruses

The influenza viruses A/California/04/09 (pH1N1)^58^, A/PR8/34 (H1N1) and A/Philippines/2/82 (H3N2) (provided by International Vaccine Institute) were grown in the allantoic cavities of 10-day-old embryonated chicken eggs for 48 h at 37 °C. The viruses were harvested from the allantoic fluid and stored at −70 °C until use. All viral experiments were performed under biosafety level 3+ (BSL3+) conditions.

### Immunization and viral challenge

Animals were randomly distributed with more than five mice or three ferrets per group. C57BL/6 mice were intramuscularly (i.m.) immunized with 10 μg of OVA plus 800 μg of either alum (Imject alum; Thermo) or PC nanogel on days 0, 14, and 21. Sera and spleens were collected on days 28 after the initial vaccination. In a separate experiment, mice were i.m. immunized with the pH1N1 vaccine containing 0.2 μg of HA with or without 800 μg of an adjuvant (alum or PC nanogel) on days 0 and 14. Sera were collected on days 14 and 28 after the initial vaccination. Splenocytes were harvested at 28 days after the initial vaccination. Two weeks after the final immunization, the mice were challenged intranasally (i.n.) with 10 LD_50_ (i.e., 6.5 log_10_ EID_50_/mL) of A/California/04/09 virus or 5 LD_50_ (i.e., 5.7 log_10_ EID_50_/mL) of A/Philippines/2/82 virus. Survival and body weight were monitored for 14 days after the viral challenge. Mice that lost greater than 25% of their body weight were considered to have reached the experimental end point and were sacrificed.

Ferrets were i.m. immunized with the pH1N1 vaccine containing 3.5 μg of HA with or without 30 mg of an adjuvant on days 0 and 14. Two weeks after the final immunization, the ferrets were challenged i.n. with 1 mL of 6 log_10_ EID_50_/mL A/California/04/09 virus or A/Philippines/2/82 virus. Sera were collected on days 14 and 28 after the initial immunization.

### Immunofluorescence/confocal microscopy

To examine antigen uptake and processing, 1 μg/mL DQ-OVA (Molecular Probes), which is OVA conjugated to a self-quenched dye that emits green fluorescence upon degradation, was mixed with 400 μg/mL of an adjuvant and incubated for 16 h at 4 °C. DCs were plated onto μ-Slides (Ibidi GmbH) and stimulated with the mixture for 4 h at 37 °C followed by incubating with LysoTracker and DAPI (Molecular Probes). To examine the accumulation of MHC-I in acidic compartments, DCs were incubated with a mixture of OVA-Texas red (1 μg/mL; Molecular Probes) with either alum or PC nanogel for 3 h at 37 °C and then exposed to LysoTracker for the last 30 min. The cells were fixed, permeabilized and stained with an anti-MHC-I (BD) followed by Alexa488-conjugated anti-mouse IgG antibody (Invitrogen). Nuclei were counterstained with DAPI. The stained cells were examined with an LSM5 Pascal confocal fluorescence microscope (Carl Zeiss). The percentages of cells in which MHC-I colocalized with LysoTracker or OVA (Manders’ coefficient) in the insets were calculated using the JACoP plugin of the ImageJ software.

### Flow cytometry

To measure various parameters of the proliferation and activation of CD8^+^ T cells, DCs were incubated with 100 μg/mL OVA protein (Sigma) combined with 200 μg/mL adjuvant for 6 h. OVA_257–264_-specific CD8^+^ T cells (1 × 10^5^ cells), selected in splenocytes from OT-I mice using a CD8^+^ T cell isolation kit (Miltenyi Biotec), were cocultured with the primed DCs (2 × 10^4^ cells) for an indicated day. After blocking with anti-CD16/CD32, the cells were stained with APC-conjugated anti-CD8a, PE-conjugated anti-CD69, and FITC-conjugated anti-CD25 (BD). For measuring T cell proliferation, carboxyfluorescein diacetate succinimidyl ester (CFSE; Molecular Probes)-labeled OT-I CD8 T^+^ cells were cocultured with the DCs. To examine cellular immunity *in vivo*, splenocytes of the immunized mice were re-stimulated with OVA_257–264_ peptide (5 μg/mL; Anaspec) in the presence of Golgiplug for 12 h. The frequencies of IFN-γ producing CD8^+^ T cells were evaluated using fluorescent-conjugated anti-CD8a, anti-IFN-γ, and a cytofix/cytoperm kit (BD). The frequency of CD8^+^ T cells expressing OVA_257–264_-specific T cell receptors (TCRs) were determined in the splenocytes by staining with fluorescence-conjugated H-2K^b^ OVA_257–264_ tetramer (MBL) and anti-CD8a (Abcam). Fluorescence compensation was optimized using cells individually labelled with each fluorochrome-conjugated antibody. Unstained cells were used to compensate for background autofluorescence. All data was gated on the live population based on cell size and morphology to eliminate cell debris and dead cells. The cells were acquired on a FACSCalibur flow cytometer (BD) and analyzed using FlowJo software (Tree Star).

### BrdU cell proliferation assay

OT-I CD8^+^ T cells were cocultured with DCs that had been exposed to OVA protein plus PC nanogel or alum for 3 days, as described above, and cell proliferation was examined with a BrdU incorporation assay kit (Roche).

### Enzyme-linked immunosorbent assay (ELISA)

Cytokine levels were measured in the culture supernatants using OptEIA kits (BD). To determine antigen-specific antibody titers, ELISA plates were coated with OVA protein (2–10 μg/mL) or the vaccine antigen (0.5 μg/mL), and ELISA was performed as described previously^11^,^23^.

### ELISPOT assay

The frequencies of IFN-γ-producing cells in the spleen were evaluated using mouse IFN-γ ELISPOT kits (BD). Spots were counted using an ELISPOT plate reader (Cellular Technology Ltd) as described previously^11^,^23^.

### HI titer assay

To determine the serum HI titers against the A/California/04/09 or A/Philippines/2/82 viruses, 25 μL of each serum was treated with 25 μL of receptor-destroying enzyme (RDE; Denka Seiken) for 16 h at 37 °C. The enzyme was heat-inactivated for 30 minutes at 56 °C, and the reactions were diluted 1:10 with PBS. The RDE-treated sera were serially diluted (1:2 dilution) in round-bottom 96-well plates, treated with 4-HA units of virus, and incubated for 30 min. Turkey red blood cells (tRBCs; 0.5% in PBS) were added, and the contents were mixed and then left to settle for 30 min. The HI titers were calculated by determining the highest dilution of each serum that inhibited virus-mediated hemagglutination of the tRBCs.

### CTL assay

Ten days after the last immunization, lung tissues were minced, enzymatically digested with collagenase D (Roche) for 1 h at 37 °C. After lysing red blood cells, CD8^+^ T cells were positively selected using a CD8^+^ T cell isolation kit (Miltenyi Biotec), cocultured with UV-inactivated A/California/04/09 virus (1 × 10^4^ TCID_50_) for 4 days, and then used as effector cells. The target EL4 cells were infected with A/California/04/09 virus (1 × 10^3^ TCID_50_) and mixed with serially diluted effector cells in 96-well U-bottom plates. Cytotoxicity was assessed using a CytoTox 96 Non-Radioactive Cytotoxicity Assay (Promega) as previously described^59^.

### Virus titration

On days 3, 5, 7 and 9 post-challenge, mouse lung samples were collected and homogenized in 1 mL of PBS containing penicillin G (Calbiochem), streptomycin (Sigma), and gentamicin (GibcoBRL). On days 3, 5, 7 and 10 post-challenge, ferret nasal wash samples were collected by the slow introduction and subsequent withdrawal of 1 mL of PBS with antibiotics. BSA (7.5%; Sigma) was added at a ratio of 1:20 (v/v) as a stabilizing agent, and the nasal wash supernatants were serially diluted and inoculated into 10-day-old embryonated chicken eggs. The viral titers were determined as described by Reed and Muench^60^ and expressed as log_10_ EID_50_/mL.

### Immunohistochemistry

On day 5 after the viral challenge, ferret lung tissues were excised from the middle lobes, fixed, and embedded in paraffin. Tissue slides were stained with a FITC-conjugated anti-influenza A NP (Thermo) antibody followed by DAPI, and then observed with a confocal fluorescence microscope.

### Depletion of CD8^+^ or CD4^+^ T lymphocytes

On days 27, 29, and 31 after the start of the above-described vaccination protocol, mice were intraperitoneally injected with 300 μg of purified anti-CD8, anti-CD4, or an isotype control antibodies (BioLegend). At one day after the final administration of depletion antibodies, cells were isolated from spleen, lymph nodes, or bone marrow of the mice. After blocking with anti-CD16/CD32, the cells were stained with FITC-conjugated anti-CD8 and PE-conjugated anti-CD4 antibodies (BD Bioscience). The expression of CD8 and CD4 was determined in the stained cells using flow cytometry.

### Statistics analyses

Statistical differences between two groups were assessed using the two-tailed Student’s *t-*test, and differences among multiple groups were assessed using a one-way ANOVA followed by Bonferroni’s correction (ANOVA/Bonferroni). The data were represented as the means ± standard deviation (SD). The log-rank test was used to analyze survival between two groups. All analyses were performed using PRISM software (GraphPad), and *P* values less than 0.05 were considered to be statistically significant.

## Additional Information

**How to cite this article**: Yang, J. *et al*. Poly-γ-glutamic acid/chitosan nanogel greatly enhances the efficacy and heterosubtypic cross-reactivity of H1N1 pandemic influenza vaccine. *Sci. Rep.* **7**, 44839; doi: 10.1038/srep44839 (2017).

**Publisher's note:** Springer Nature remains neutral with regard to jurisdictional claims in published maps and institutional affiliations.

## Supplementary Material

Supplementary Information
