# Supplementary material for: Poly-γ-glutamic acid/chitosan nanogel greatly enhances the efficacy and heterosubtypic cross-reactivity of H1N1 pandemic influenza vaccine
Source: Sci Rep. 2017 Mar 21;7:44839. doi: 10.1038/srep44839 (PMC5359587; doi:10.1038/srep44839)
Supplement: Supplementary Information [file srep44839-s1.pdf]

Supplementary Information

Poly-γ-glutamic acid/chitosan nanogel greatly enhances the efficacy and heterosubtypic cross-reactivity of H1N1 pandemic influenza vaccine

Jihyun Yang[1†], Sang-Mu Shim[2,3†], Quyen Nguyen Thi[1], Eun-Ha Kim[2,4], Kwang Kim[5], Yong-Taik Lim[6], Moon-Hee Sung[5,7], Richard Webby[8] and Haryoung Poo[1,2*]

[1]Microbiomics and Immunity Research Center, Korea Research Institute of Bioscience and Biotechnology, Daejeon, Republic of Korea.[2]Viral Infectious Disease Research Center, Korea Research Institute of Bioscience and Biotechnology, Daejeon, Republic of Korea. [3]Department of Molecular Biology and the Institute for Molecular Biology and Genetics, Chonbuk National University, Jeonju, Republic of Korea. [4]College of Medicine, Chungbuk National University, Chengju, Republic of Korea.[5]BioLeaders Corporation, Daejeon, Republic of Korea. [6]Department of Chemical Engineering, Sungkyunkwan University Advanced Institute of Nanotechnology, Suwon, Republic of Korea.[7]Department of Bio and Nanochemistry, Kookmin University, Seoul, Republic of Korea. [8]Division of Virology, Department of Infectious Diseases, St. Jude Children's Research Hospital, Memphis, Tennessee, USA.

[†]These authors contributed equally to this work.

[*]To whom correspondence should be addressed:

Haryoung Poo, PhD

Microbiomics and Immunity Research Center, Korea Research Institute of Bioscience and Biotechnology, Daejeon, 305-806, Republic of Korea

e-mail: haryoung@kribb.re.kr,Tel: +82-42-860-4157, Fax: +82-42-879-8498

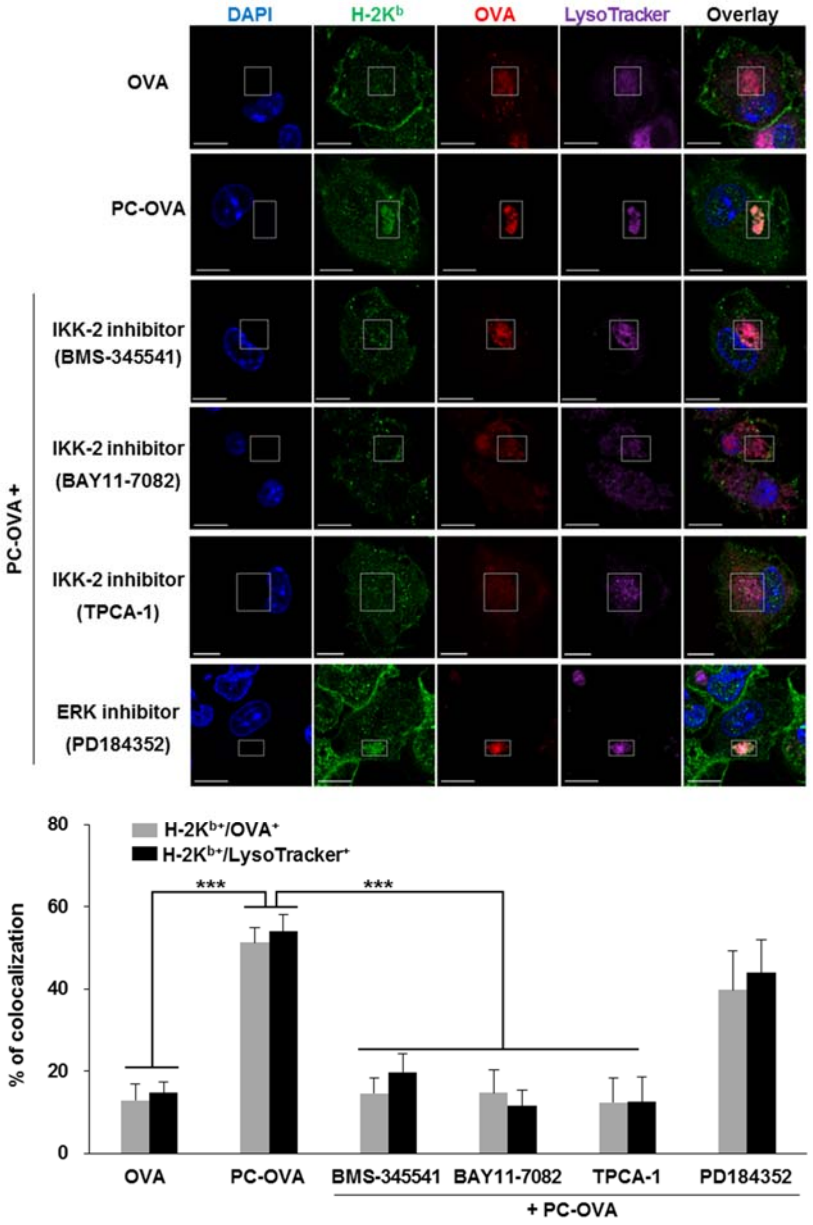

Supplementary Figure 1. IKK-2 signaling is important to enhance the antigen peptide-MHC-I complex for cross-presentation. DCs were pre-incubated with IKK-2 inhibitors (BMS-345541, BAY 11-7082, and TPCA-1) or an ERK inhibitor (PD184352) for 1 h, and then further incubated with OVA-Texas red in the presence of PC nanogel for 3 h. The cells were stained with fluorescent-conjugated anti-MHC-I followed by further incubation with LysoTracker, and observed by fluorescence microscopy. Scale bar, 10 µm. Bar graphs indicate the percent of colocalization in the boxed insets (Manders' coefficient; n = 20 cells). *, $P < 0.05$; **, $P < 0.01$; and ***, $P < 0.001$.

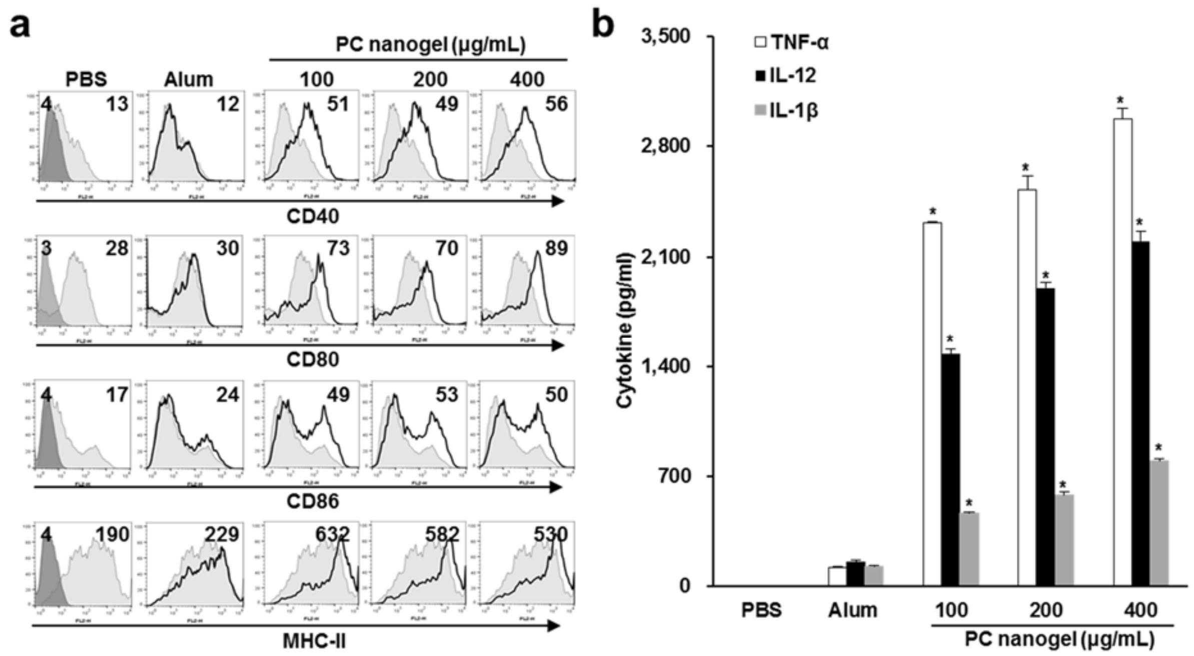

Supplementary Figure 2. PC nanogel induces innate immune responses. (a) DCs were incubated with alum or PC nanogel for 24 h, and the surface expression levels of CD40, CD80, CD86, and MHC-II were analyzed by flow cytometry. The dark and bright grey areas indicate an isotype control and a negative control (PBS treatment), respectively. MFI is shown in each histogram: the right one indicates protein expression, and the left one, isotype control. (b) The concentrations of TNF-α, IL-12, and IL-1β in the culture supernatants were determined by ELISA. Statistically significant differences between the PC nanogel group and the other groups are indicated as follows: *, $P < 0.05$; **, $P < 0.01$; and ***, $P < 0.001$. Data are presented as the mean ± standard deviation (SD), and representative of three independent experiments with similar results.

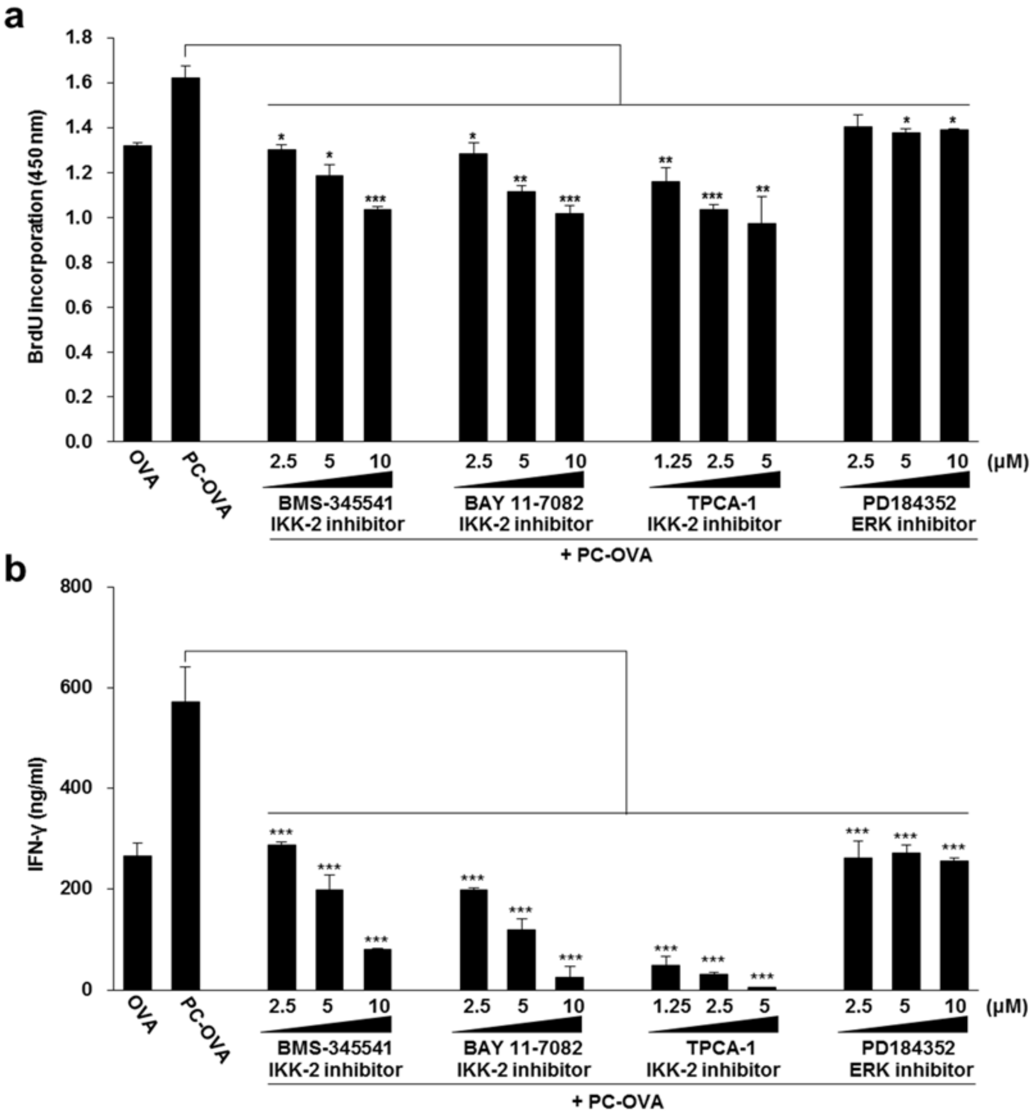

Supplementary Figure 3. PC nanogel-induced CTL activities are mediated by IKK-2 signaling. DCs were pre-incubated with IKK-2 inhibitors (BMS-345541, BAY 11-7082, and TPCA-1) or an ERK inhibitor (PD184352) for 1 h, and then pulsed with OVA combined with PC nanogel for 6 h. The pulsed DCs were cocultured with OT-I $CD8^+$ T cells for 3 days. (a) T cell proliferation was determined by BrdU incorporation assays and (b) the concentrations of IFN-γ in the culture supernatants were determined by ELISA. Statistically significant differences between the PC nanogel group and the inhibitor-treated groups are indicated as follows: *, $P < 0.05$; **, $P < 0.01$; and ***, $P < 0.001$. Data are presented as the mean ± SD, and representative of three independent experiments with similar results.

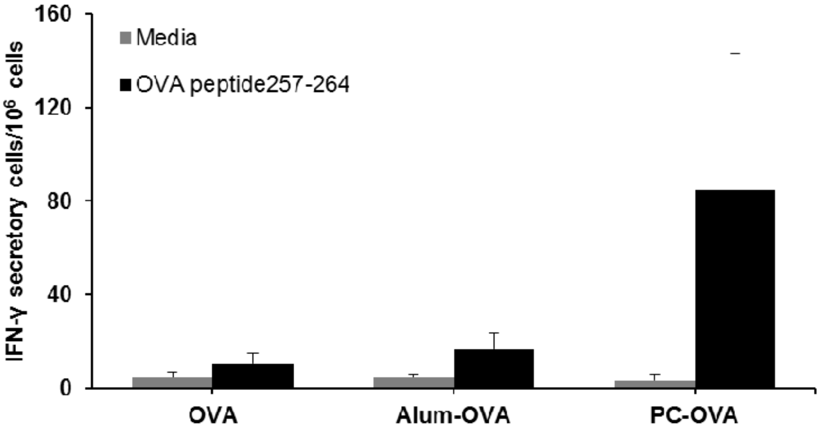

Supplementary Figure 4. PC nanogel-adjuvanted OVA induces long-lasting cellular immune responses. C57BL/6 mice (n = 3) were i.m. immunized on days 0, 14, and 21 with 10 μg of OVA mixed with alum or PC nanogel. At 24 weeks after the final immunization, splenocytes of immunized mice were re-stimulated with $OVA_{257-264}$ peptide and subjected to enumeration of IFN-γ-secreting cells by ELISPOT assay.

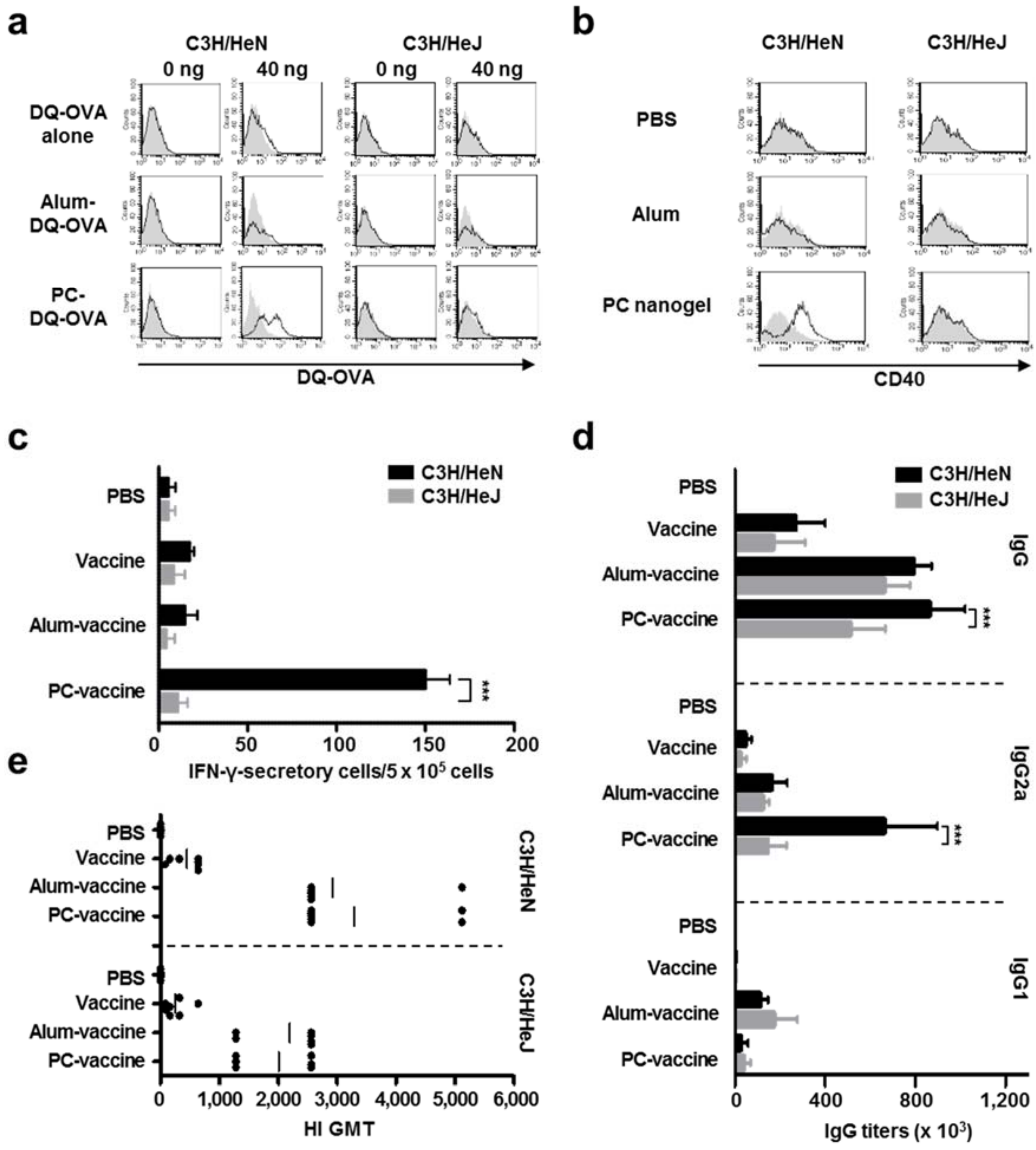

Supplementary Figure 5. The adjuvant effects of PC nanogel are TLR4-dependent. (a and b) DCs were prepared by culturing bone marrow cells from C3H/HeN (wild-type) and C3H/HeJ (TLR4-deficient) mice (n = 5) with GM-CSF and IL-4. (a) The DCs were incubated with DQ-OVA with or without adjuvants (i.e., alum or PC nanogel), and fluorescent intensity of DQ-OVA was analyzed by flow cytometry. (b) DCs were stimulated with alum or PC nanogel for 24 h, and the surface expression level of CD40 was analyzed by flow cytometry. The grey area indicates an isotype control. (c-e) C3H/HeN and C3H/HeJ mice were

immunized intramuscularly with 0.2 μg of pandemic vaccine. (c) Splenocytes form the immunized mice were isolated and re-stimulated with inactivated pH1N1 virus, and IFN-γ-producing T cells were assessed by ELISPOT assays. (d) The serum titers of influenza vaccine-specific IgG, IgG2a, and IgG1 were determined by ELISA. (e) Serum HI antibody titers against the homologous virus were measured, and are presented as individual and HI GMT titers. Statistically significant differences between C3H/HeN and C3H/HeJ mice are indicated as follows: *, $P < 0.05$; **, $P < 0.01$; and ***, $P < 0.001$. Data are presented as the mean ± SD, and representative of two independent experiments with similar results.

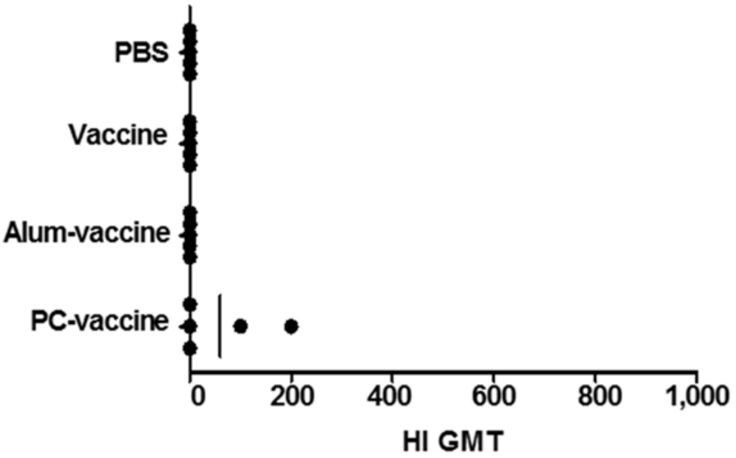

Supplementary Figure 6. Neutralizing humoral immunity is barely associated with PC nanogel-induced protection against heterosubtypic H3N2 virus infection. Sera of vaccinated ferret were assessed to measure HI antibody titers against a heterosubtypic H3N2 virus. Results are shown as individual and HI GMT titers.
